# Chronic Fatty Acid Exposure Disrupts SH-SY5Y and Neuronal Differentiation and Is a Potential Link Between Type-2 Diabetes and Alzheimer’s Disease

**DOI:** 10.1007/s12035-025-05367-6

**Published:** 2025-11-27

**Authors:** Imogen L. Targett, Kate Pring, Ana I. Martinez Valiente, David Qualtrough, Myra E. Conway, Lucy A. Crompton, Tim J. Craig

**Affiliations:** 1 Centre for Research in Biosciences, School of Applied Sciences, University of the West of England, Coldharbour Lane, Frenchay, Bristol, BS16 1QY UK; 2 University of Derby, Derby, DE22 1GB UK

## Abstract

Alzheimer’s disease (AD) is the most common cause of dementia, for which there is no curative treatment and few disease-modifying therapies. The vast majority of AD is late onset (LOAD) without a specific genetic cause, although many genetic and non-genetic risk factors have been identified. One of the most significant modifiable risk factors is diet/lifestyle, with type-2 diabetes mellitus (T2DM) increasing LOAD risk by over 50%. Despite the epidemiological data, the reasons for this link are not understood. Here, we investigated whether altered free fatty acid (FFA) levels seen in T2DM can adversely affect neuronal differentiation, a crucial stage in adult hippocampal neurogenesis (AHN), which is defective in LOAD. We show that chronic exposure of the neuroblastoma cell line, SH-SY5Y to T2DM-relevant levels of the FFAs, oleate and palmitate, profoundly affects the differentiation of these cells. This effect is particularly pronounced for the saturated FFA, palmitate, resulting in neuronal cells of altered morphology, lacking expression of key synaptic markers. We further demonstrate that this exposure dysregulates insulin signalling, GSK3β activity, CDK5 levels and CREB phosphorylation. Crucially, these effects were only observed on exposure during differentiation and can be partially replicated in hiPSC-derived forebrain neurones. Although APP expression is increased by palmitate exposure, there was no increase in secreted or intracellular Aβ, and tau phosphorylation was reduced, implying that these defects are separate from the classical hallmarks of AD. We conclude that long-term, chronic exposure of differentiating neurones induces pathological changes that may explain the link between T2DM and LOAD.

**Supplementary Information:**

The online version contains supplementary material available at 10.1007/s12035-025-05367-6.

## Introduction

Neurodegenerative diseases are amongst the most pressing public health concerns, both in terms of public impact and economic costs. In many countries, the incidence of these diseases is increasing, partly due to an ageing population, but also potentially precipitated by changing diets and lifestyles. The most common neurodegenerative disease is Alzheimer’s disease (AD), accounting for approximately 65% of all cases worldwide (according to the WHO 2024). AD is characterised by the key pathological hallmarks of extracellular amyloid plaques, composed of aggregated β-amyloid (Aβ) peptides, and intracellular neurofibrillary tangles (NFTs), composed of paired-helical filaments of hyperphosphorylated tau (a microtubule binding protein) [1]. There is still uncertainty as to the precise pathological roles of these aggregates; however, one model is that the formation of Aβ oligomers is an early stage of AD (potentially even prodromal) [2]. Aβ oligomers then precipitate later pathological events such as tau hyperphosphorylation and synaptic dysfunction, ultimately leading to neuronal dysfunction and loss and causing the characteristic symptoms of short-term memory loss, cognitive impairment and ultimately senile dementia [3]. It should be noted, however, that some studies have concluded that, although this ‘amyloid first’ model holds true in many cases, there are also patients who display a ‘tau first’ pathology [4].

Both tau and Aβ have physiological as well as pathophysiological roles: tau is a brain-specific microtubule-binding protein [5], whose phosphorylation is associated with neuronal development [6]; Aβ is derived from the proteolytic processing of the amyloid precursor protein (APP) by β- and γ-secretases and has several ascribed roles including neuronal development/differentiation, neuroprotection and synaptic plasticity [7]. The early, prodromal events in AD are poorly understood, simply because they are likely to occur years or even decades prior to the onset of symptoms [8]. Furthermore, animal models are almost exclusively based on human mutations which cause early onset AD, a form that only represents 5% of all cases [1]. The vast majority of AD cases are ‘sporadic’ or ‘late onset’ and have no defined genetic cause (late onset Alzheimer’s disease (LOAD)). For this reason, the early events in AD that lead to APP processing switching from being physiological to pathological are unknown.

Insight, however, can be gained from identified risk factors. Of particular interest is the relatively recent observation that type-2 diabetes mellitus (T2DM) increases the risk of LOAD by 50–75% [9–11]. Although there is a genetic element to T2DM, the most significant risk factors appear to be diet- and lifestyle-related [12]; indeed, there is increasing evidence that high-fat diets and low levels of exercise, especially in late middle age, are also risk factors for late-onset AD [13, 14], and that the AD brain displays insulin resistance [15]. For this reason, there has been a lot of focus in recent years on neuronal metabolism and the role of insulin in the brain. Indeed, insulin has now been demonstrated to have many neuronal roles, including neuronal survival [16, 17], hippocampal synaptic plasticity [18, 19] and potentially neurogenesis [20]. This latter observation is particularly interesting as one of the first regions of the brain to be affected in AD, the hippocampus, is also one of the only sites of adult neurogenesis (termed adult hippocampal neurogenesis or AHN) [21], raising the prospect that dysfunctional neurogenesis may represent an early stage of LOAD. Indeed, several studies have now demonstrated that hippocampal neurogenesis is reduced in the AD brain [22, 23], and that stimulating this process can alleviate dementia-like symptoms in mouse models of AD [24]. Despite all these data, the links between T2DM and AD remain poorly understood. A possible link between T2DM and LOAD is that elevation of serum free fatty acid (FFA) levels (which occurs both in T2DM and obesity [25, 26]) interferes with normal hippocampal neurogenesis and precipitates the early stages of LOAD. Indeed, several studies have implicated FFAs in the elevation of AD markers [27] and modulating embryonic neurogenesis [28], making them a good candidate for the link between T2DM, AD and neurogenesis.

For this reason, we decided to study the effects of chronically elevated FFAs on neuronal differentiation and development, mimicking the chronically elevated levels of FFAs which persist over a long period of time in T2DM. Unlike several previous studies (e.g. [28–30]), we have employed long-term exposure with levels of fatty acids seen in the CSF (and presumably therefore in the brain) of obesity and T2DM patients [31], which are lower than those traditionally used in lipotoxicity experiments. For our studies, we used the neuroblastoma cell line, SH-SY5Y as our model of neuronal differentiation [32, 33]. We demonstrate a profound alteration of many cellular signalling pathways by chronic exposure to free fatty acids during differentiation, an effect which is more pronounced, but not limited to, saturated fatty acids. These alterations resulted in dysfunctional synaptogenesis, insulin resistance and dysregulation of critical neuronal signalling pathways. Interestingly, however, there was no increase in either Aβ production (despite an increase in APP expression induced by FFAs) or tau phosphorylation (which was actually reduced by elevated FFAs), suggesting that this effect might represent an early, prodromal stage of LOAD. Based on these data, we propose that the link between T2DM and AD is at least partly due to the exposure of differentiating neurones to elevated fatty acids.

## Materials and Methods

### SH-SY5Y Cell Culture and Differentiation

Human SH-SY5Y neuroblastoma cells were obtained from American Type Culture Collection (ATCC, Manassas, VA). The cells were cultured in Dulbecco’s modified Eagle’s medium F-12, glutamax supplement (DMEM/F-12; Gibco, Grand Island, NY, 11524436) supplemented with 10% foetal bovine serum (FBS; Gibco, 11514436) and 1% non-essential amino acids (NEAAs; Gibco, 11140050) and maintained in a humid environment at 37 °C and 5% CO2 atmosphere. Media were replaced twice weekly, and cells were subcultured when they reached 70–80% confluency.

The SH-SY5Y differentiation protocol was followed as previously described in [33]. Briefly, cells were seeded at 10,526 cells/cm^2^ in DMEM/F-12 with 10% FBS and 1% NEAAs (day 1) in 12-well or 24-well plates. On day 0, the medium was replaced with diff1 media (DMEM/F-12, 2% FBS, 1% NEAAs, 10 µM retinoic acid (RA)) after a 1 × phosphate buffer saline (PBS) wash. On days 3 and 6, cells were washed and given diff2 media (DMEM/F-12, 1% FBS, 1% NEAAs, 10 µM RA, 50 ng/ml brain-derived neurotrophic factor (BDNF)).

### hIPSC Cell Culture and Differentiation

The hiPSC (nas2) cell line was obtained from the Devine lab [34], and forebrain neural stem cells (fbNSCs) were generated in Jon Lane’s lab (University of Bristol) [35–37]. fbNSCs were maintained in N2B27 medium containing: DMEM/F-12, neurobasal (Gibco, 21103049), B27 (Gibco, 17504044), N2 (Gibco, 17502048), insulin (Sigma, I9278), NEAAs, glutamax (Gibco, 35050038) and 2-mercaptoethanol (Gibco, 31350010).

Once the cells reached 95% confluence, cells were passaged onto 1 × geltrex-coated (Gibco, 12063569) 6 or 12 well plates and treated with Y-27632 ROCK inhibitor (Medchem, 16474058) before and after splitting. On day 32, cells were split onto geltrex-coated 12 or 24 well plates with N2B27 medium for Western blot or immunocytochemistry (ICC) analysis. DAPT (Tocris, 2634) was added to the cells post-splitting to promote neuronal differentiation. Fatty acids were added at this point so that they were present throughout the differentiation process. Media were changed every 3–4 days, and fatty acid treatment was added after each media change. Full differentiation was achieved in 10 days.

### Fatty Acid Treatments

100 mM stock solutions of palmitic acid (Merck, P0500) and oleic acid (Merck, O1008) were prepared. Fatty acids (FAs) were prepared in glass vials with 100% ethanol and dissolved by heating at 50 °C for 10 min. Sterile fatty-acid-free BSA (Merck, 03117057001) was prepared at 2 mM with molecular biology grade water. FAs were conjugated to 2 mM BSA at a 1:5 molarity ratio, to make a final concentration of 10 mM. Vehicle control was 2 mM BSA with 10% ethanol, i.e. all components from the fatty acid treatment apart from the fatty acid, added at the same volume as the relevant fatty acid treatment. Therefore, control cells were exposed to the same concentration of BSA and ethanol as the fatty acid-treated cells. The vials were placed in a shaking incubator at 225 rpm, 37 °C for 4 h and stored at −20 °C until required. Fatty acid treatment was added after every media change throughout the 10-day time course (i.e. starting at D0).

### Western Blotting

SH-SY5Y and fbNSCs cells were lysed in ice-cold lysis buffer (25 mM Hepes, 150 mM NaCl, 0.1% SDS, 1% triton, phosphatase/protease inhibitor tablet, pH 7.4) as described above. Samples were centrifuged at 20,000 × g for 5 min at 4 °C, and the supernatant was stored at −20 °C. Lysates’ protein concentration was determined using a detergent compatible Bradford assay (Thermofisher, 15398086) and diluted to equal concentrations. Samples were combined with Laemmli loading buffer and heated at 75 °C for 5 min before resolution on sodium dodecyl sulphate polyacrylamide gel electrophoresis (SDS-PAGE) and transferred to polyvinylidene fluoride (PVDF) membrane. Blots were blocked with 5% milk and incubated with primary antibodies (Table 1) at 4 °C overnight. The blots were washed with 1 × tris-buffered saline-tween 20 (TBST; 10 mM Tris–HCl (pH 7.6), 150 mM NaCl and 0.1% Tween-20) and incubated with the appropriate secondary antibodies (Table 2) for 1–2 h in the dark at RT. The blots were imaged with a LiCor Odyssey Fc, using either HRP substrate (for proteins of interest) or 800 nm fluorescent secondary antibodies (for GAPDH), and bands were quantified using LICOR Image Studio software. For normalisation, the density of each protein band was normalised to the equivalent glyceraldehyde 3-phosphate dehydrogenase (GAPDH) density from the same lane. Values within each experimental replicate were further normalised to the mean of all normalised values within that replicate to allow direct comparison between blots whilst preserving variability.

**Table 1:** List of primary antibodies for Western blotting and immunocytochemistry

Antibody	Species	Company	Cat number	Dilution
β-III tubulin (D71G9)	Rabbit	Cell Signaling technologies	5568	1:2000
Anti-β III tubulin antibody (2G10)	Mouse	Abcam	ab78078	1:700 ICC
Synaptophysin (D8F6H)	Rabbit	Cell Signaling technologies	36406	1:20,000 WB, 1:500 ICC
PSD95-Specific, DLG4	Rabbit	Protein Tech	20665–1-AP	1:500
Recombinant Anti-Choline Acetyltransferase (ChAT)	Rabbit	Abcam	ab181023	1:1000
AKT	Mouse	Protein Tech	60203-2-Ig	1:2000
Phospho-AKT1 (Ser473)	Mouse	Protein Tech	80462-1-RR	1:1000
Phospho-p44/42 MAPK (Erk1/2) (Thr202/Tyr204)	Rabbit	Cell Signaling technologies	4377	1:1000
GSK-3β (27C10)	Rabbit	Cell Signaling technologies	9315	1:1000
Phospho-GSK3B (Ser9)	Mouse	Protein Tech	67558-1-Ig	1:2000
p35/25 (C64B10)	Rabbit	Cell Signaling technologies	2680	1:1000
Recombinant Anti-Tau (phospho S396)	Rabbit	Abcam	ab109390	1:2000
Anti-Tau (phospho S202 + T205)	Rabbit	Abcam	ab210703	1:1000
Tau (D1M9X) XP®	Rabbit	Cell Signaling technologies	46687	1:1000
APP	Rabbit	Protein Tech	27320-1-AP	1:1000
BACE1	Rabbit	Abcam	ab263901	1:1000
Phospho-CREB (Ser133) (87G3)	Rabbit	Cell Signaling technologies	9198	1:1000 WB, 1:500 ICC
CREB (48H2)	Rabbit	Cell Signaling technologies	9197	1:1000
Anti-Syntaxin antibody [STX01 (HPC-1)]	Mouse	Abcam	ab3265	1:100
CDK5 (1H3)	Mouse	Cell Signaling technologies	12134	1:500
SNAP-25	Rabbit	Protein Tech	14903-1-AP	1:2000
Glyceraldehyde 3-phosphate dehydrogenase (GAPDH)	Mouse	Santa Cruz biotechnologies	sc47724	1:250

**Table 2:** List of secondary antibodies for Western blotting and immunocytochemistry

Western blotting	Company	Cat number	Dilution
Goat anti-Rabbit IgG (H + L) Secondary Antibody, HRP	Invitrogen	15217664	1:10,000
Goat anti-Mouse IgG (H + L) Cross-Adsorbed Secondary Antibody	Invitrogen	15291378	1:10,000
IRDye 800CW Donkey anti-Mouse	Li-cor Biosciences	925–32212	1:20,000
**ICC**			
Goat anti-Rabbit IgG (H + L) Highly Cross-Adsorbed Secondary Antibody, Alexa Fluor™ 488	Invitrogen	10236882	1:1000
Goat anti-Mouse IgG (H + L) Highly Cross-Adsorbed Secondary Antibody, Alexa Fluor™ 568	Invitrogen	10226882	1:1000

### Immunocytochemistry

SH-SY5Y cells were seeded onto 0.25 × geltrex-coated coverslips at a density of 10,526 cells/cm^2^. Following differentiation, cells were fixed with 4% paraformaldehyde for 15 min at RT, washed with 1 × PBS and stored at 4 °C until required. Coverslips were blocked/permeabilised for 1 h with blocking solution (10% horse serum, 1% BSA in 0.1% PBTx (PBS + 0.1% Triton-X)). Cells were incubated overnight on droplets of primary antibody (Table 1) in primary solution (1% horse serum, 0.1% BSA in 0.1% PBTx) in a humid chamber with 1 × PBS at 4 °C. Cells were washed three times with 1 × PBS and incubated with secondary fluorescent antibodies in primary solution for 1–2 h at RT (Table 2). For lipid staining, the secondary antibody was added to lipid spot 610 stain solution instead of primary solution (Biotium, BT70069-T). Coverslips were washed 3 × with 1 × PBS and co-stained with DAPI solution then mounted onto slides in Mowiol 40–88 mounting media (Merck, 324590) All quantification was performed using ImageJ, with each biological replicate consisting of the average of multiple ROIs of several individual cells. For neurite analysis of synaptophysin, synaptophysin signal was normalised to β-III tubulin signal. In all cases, ROIs were chosen from either the β-III tubulin (Fig. 2) or DAPI (Fig. 8) channel.

### Enzyme-Linked Immunosorbent Assay (ELISA)

SH-SY5Y cells were plated onto 12 well plates at a density of 10,526 cells/cm^2^ and were differentiated as mentioned above. On day 10, media was collected from cells and the cells were lysed with ice-cold RIPA buffer with protease inhibitors. Lysates were centrifuged at 20,000 × g for 5 min at 4 °C, and supernatant was collected for detecting human amyloid-β_1–42_ (Aβ42). Media was concentrated using centrifugal concentrators (Sartorius, VS0191). Aβ42 levels were assessed using an ELISA kit (Bio-techne, DAB142) following the manufacturer’s protocol. Standards were prepared and the plate was washed twice with wash buffer. One hundred microlitre sample or standards were added per well and incubated for 2 h at 4 °C. Wells were washed 4 times, and 200 µl of cold human Aβ42 conjugate was added to each well for 2 h at 4 °C. Wells were washed again, and 200 µl of substrate solution was added to the wells for 30 min at RT in the dark. Fifty micolitres of stop solution was added to each well, and readings were taken using a Vantastar plate reader at 450 nm. 

### MTS Assay

MTS assays were performed using CellTiter96 Aqueous One solution (Promega) according to the manufacturer’s instructions. Briefly, cells were plated in a 96-well plate at the density above and differentiated as described. At day 10, media was aspirated and replaced with 100 µl fresh medium. Twenty microlitres of MTS reagent was added and incubated at 37 °C for 150 min, before absorbance was read at 495 nm. Results were normalised to control (untreated) cells and expressed as a percentage.

### Quantitative PCR

The Taqman Cells-to-CT kit was used to analyse mRNA from differentiated SH-SY5Y cells using the manufacturer’s protocol (ThermoFisher, 4399002). Briefly, on day 10 of SH-SY5Y differentiation, cells were lysed in lysis solution and stored at –80 °C until required. DNase I was added to the sample for 5 min at RT; stop solution was added for 2 min at RT and then placed on ice. Lysates were reverse transcribed to synthesise cDNA and run on a RT thermal cycler programme. The specific gene expression assays used were as follows: RNA binding protein, fox-1 homolog 3 (NeuN/Rbfox3; Assay ID: Hs01370654_m1), doublecortin (DCX; Assay ID: Hs00167057_m1), metastasis associated lung adenocarcinoma transcript 1 (MALAT1; Assay ID: Hs00273907_s1) and β-actin (Assay ID: Hs00357333_g1). The cDNA samples were added to wells of a real-time PCR plate, and amplification was performed using a StepOne Plus PCR system. Results were analysed using StepOne software.

### Seahorse Metabolic Analysis

SH-SY5Y cells were plated in a 24-well seahorse microplate at 2 × 10^4^ per well and were subject to differentiation over 10 days. On day 10, cells were assessed using the Seahorse XF cell Mito stress kit using the manufacturer’s instructions (Agilent Technologies, Santa Clara, CA). Briefly, on day 9, cartilages were hydrated in calibrant at 37 °C in a non-CO_2_ incubator overnight. Cell media was changed to warm DMEM medium (Agilent, 103575-100) by supplementing seahorse XF base medium with 1 mM pyruvate, 2mM glutamine and 10 mM glucose and incubated at 37 °C in a non-CO_2_ incubator for 1 h. Stock compounds were prepared and loaded into the sensor cartilages as follows: 1 µM oligomycin, 2 µM FCCP and 0.5 µM rotenone/antimycin-A. The seahorse was run on the seahorse XFe24 analyser and analysed using the Wave software and Agilent mito-stress test analysis spreadsheets. 

### Statistical Analysis

All data are expressed as mean ± standard error of the mean (SEM). Statistical analyses were performed using Prism 9.5.1, and *p*-value was determined as ns, *p < 0.05, ** *p* < 0.01, ***p < 0.001 and ****p < 0.0001.

## Results and Discussion

Due to their ease of culture and well-established characteristics, we employed SH-SY5Y human neuroblastoma cells as our main model of neuronal differentiation. These cells display many characteristics of neuroblasts but can be readily differentiated using retinoic acid and BDNF [33]. Importantly, differentiated SH-SY5Y cells produced using this protocol display a cholinergic/glutamatergic phenotype [32, 33], increasing their relevance to studies of LOAD. Firstly, we further characterised this differentiation protocol using qPCR to examine the expression of key markers of neuronal differentiation (Fig. S1). These data indicate a robust increase in the expression of DCX mRNA over the 10-day time course of the protocol, but a lack of NeuN expression, indicating that this protocol likely represents the differentiation of neuroblasts to immature neurones, without progressing to the mature neurone stage [38]. We therefore concluded that this is a useful model to examine the vulnerability of early-stage neurogenesis to fatty-acid-induced damage; however, we acknowledge that results obtained cannot be fully extrapolated to *bona fide* neurones.

**Fig. 1:**
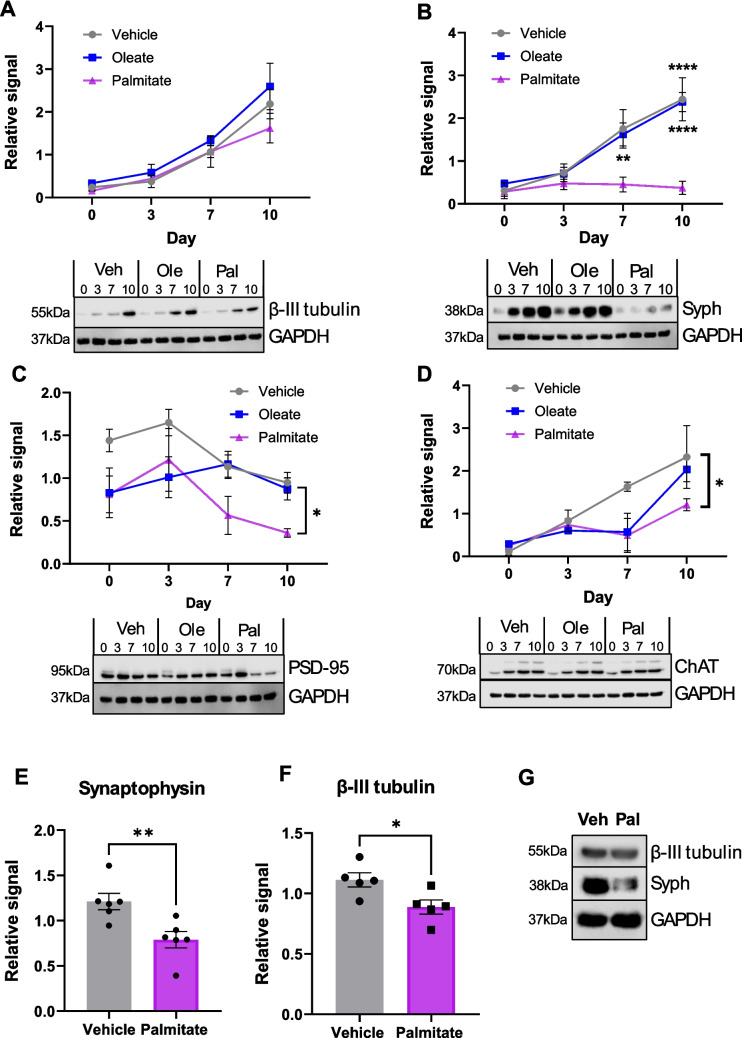
Chronic palmitate treatment induces defective synaptic differentiation in SH-SY5Y cells and hISPC-derived neurones. **A**–**D **Western blotting quantification of key differentiation markers at 4 timepoints (D0, D3, D7 and D10) during SH-SY5Y cell differentiation, treated throughout the protocol with either 20 µM BSA-conjugated palmitic or oleic acid, or an equivalent volume of vehicle. All protein levels are normalised to a GAPDH loading control and normalised within each repeat. All values are the mean of four independent repeats, ± SEM. A representative Western blot is shown below each graph. *p < 0.05, **p < 0.01 (comparing palmitate treatment to control), 2-way ANOVA with Bonferroni’s post-hoc test. **A** β-III tubulin. **B** Synaptophysin. **C** PSD-95. **D** Choline-acetyl transferase (ChAT). **E**–**G** Western blot analysis of β-III tubulin (**E**) and synaptophysin (**F**) levels of D10 differentiated hIPSC-derived forebrain neurones exposed to 20 µM FFAs. Protein levels were normalised to GAPDH loading control and normalised within each repeat. Representative blots shown in **G**. Data are the mean of 6 repeats. *p < 0.05, **p < 0.01 on Mann-Whitney unpaired *t*-test

In order to mimic the exposure of differentiating hippocampal neurones to diabetic conditions, we performed this differentiation in the presence of either 20 µM oleic acid or 20 µM palmitic acid. These are the most abundant unsaturated and saturated fatty acids (SFAs), respectively, found in human serum, and a recent study [31] has demonstrated that 20 µM accurately represents the levels of palmitic acid found in cerebrospinal fluid of patients with T2DM and obesity (compared to approx. 8 µM control patients). Control SH-SY5Y cells were exposed to an equal volume of vehicle (10% EtOH, 2 mM BSA). Lipid droplet staining using LipidSpot 610 stain demonstrated clearly that cells treated with both palmitate and oleate accumulated intracellular lipid droplets, which were considerably less apparent (although not completely absent) in control, vehicle-treated cells (Fig. S2). Additionally, FFA (especially palmitate)-exposed cells demonstrated altered morphology, with less obvious bouton-like structures along their neurites. In order to characterise differences in differentiation at a molecular level, Western blotting for key differentiation markers was performed on cell lysates at D0, D3, D7 and D10. These results demonstrated that exposure to 20 µM palmitate, but not oleate, profoundly reduced synaptophysin expression (Fig. 1B) but not beta-III tubulin expression (Fig. 1A) across the differentiation time course. PSD-95 expression was also significantly reduced by palmitate exposure (Fig. 1C), but only at D10. Interestingly, we did not see a strong increase in PSD-95 throughout differentiation in the SH-SY5Y cells, compared to synaptophysin. This is consistent with earlier observations that the cells reach an immature neurone stage but do not fully develop into mature neurones.

These data imply that neurite outgrowth is unaffected by palmitate exposure; however, both pre- and post-synaptic development are inhibited. Western blotting for choline-acetyl transferase (ChAT) showed that this protein is also unaffected apart from a slight reduction at D10 in palmitate-treated cells (Fig. 1D), suggesting that the cholinergic phenotype of the differentiated neurones is mostly unchanged. We fully appreciate that the differentiation of SH-SY5Y cells does not faithfully represent the differentiation of human neurones in vivo; therefore, we repeated this protocol using hIPSC-derived cortical neurones. Using these cells, we found that exposure to 20 µM palmitic acid during the differentiation process also significantly reduced the expression of synaptophysin in mature neurones compared to vehicle control (Fig. 1E–G). Interestingly, in the hIPSC-derived forebrain neurones, β-III tubulin expression was also reduced by palmitate exposure. These data suggest that the effect we see in SH-SY5Y cells can be partially recapitulated in hIPSC-derived neurones; however, there maybe differences in the responses of these cells. Importantly, exposure of the SH-SY5Y cells to FFAs after differentiation had no effect on the expression of either synaptophysin or PSD-95 (Fig. S3), suggesting that differentiating, not mature, cells are uniquely vulnerable to fatty-acid-induced dysfunction. We also exposed cells to 8 µM oleate or palmitate, as these are the levels found in the CSF of metabolically healthy individuals according to Melo et al*.* [31]; however, neither palmitate nor oleate at these concentrations had a significant effect on the induction of synaptophysin or β-III tubulin expression during differentiation (Fig S4).

**Fig. 2:**
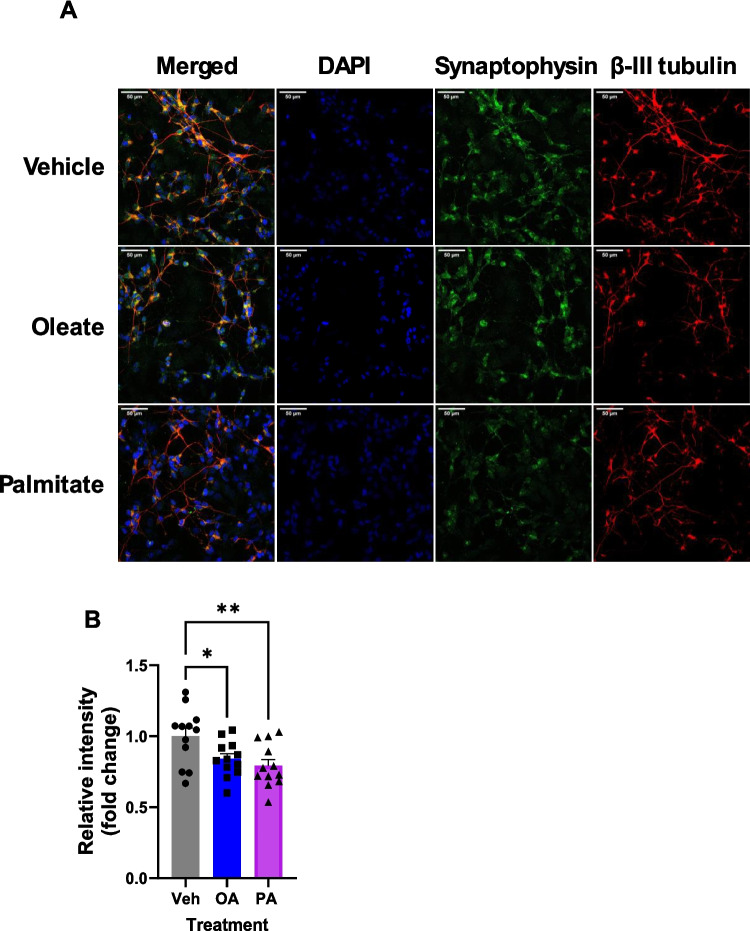
Chronic palmitate exposure during differentiation alters synaptophysin distribution in SH-SY5Y cells. **A** Representative immunocytochemistry images of β-III tubulin (red), synaptophysin (green) and DAPI staining in D10 cells following chronic FFA treatment. Scale bar = 50 µm. **B** Quantification of synaptophysin levels on neurites, normalised to levels of β-III tubulin. Data are mean of 13 independent experiments, ± SEM. *p < 0.05, **p < 0.01, 1-way ANOVA with Bonferroni’s post-hoc test

Consistent with these data, immunocytochemistry imaging of differentiated cells showed extensive neurite outgrowth in all conditions (Fig.2 A); however, quantification revealed a reduction in synaptophysin expression in these neurites in cells treated with palmitate (Fig. 2B). Taken together, these data indicate a defect in synaptogenesis induced by 20 µM palmitate exposure of cells during differentiation, implying a specific vulnerability of differentiating neurones to fatty acid-induced damage. Furthermore, these effects occur in SH-SY5Y cells and hIPSC-derived forebrain neurones, validating SH-SY5Y cells as a model for at least some aspects of differentiation of human forebrain neurones.

**Fig. 3:**
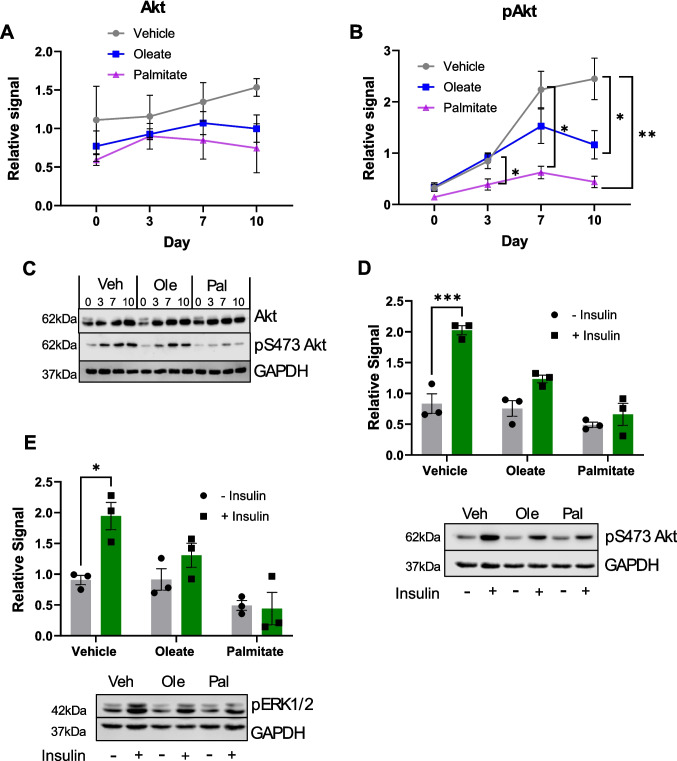
Chronic fatty acid exposure during differentiation inhibits insulin signalling in SH-SY5Y cells. **A**–**E** Western blotting quantification of Akt and ERK, either total levels or phospho-specific, in SH-SY5Y cells differentiated in the presence of 20 µM BSA-conjugated palmitic or oleic acid, or an equivalent volume of vehicle. All protein levels are normalised to a GAPDH loading control and normalised within each repeat. All values are the mean of four independent repeats, ± SEM. *p < 0.05, **p < 0.01 (comparing palmitate treatment to control), 2-way ANOVA with Bonferroni’s post-hoc test. **A** Total Akt levels at different differentiation timepoints. **B** Phospho-Akt (S473) levels at different differentiation timepoints. **C** Representative Western blots. **D** Insulin-responsive p-Akt (D10 cells lysed after 30 min pulse with 100 nm human insulin, or control), normalised to GAPDH levels. **E** Insulin-responsive p-ERK T202/Y204 (D10 cells lysed after 30 min pulse with 100 nm human insulin, or control), normalised to GAPDH levels. Representative blots for **D** and **E** shown below graphs

In order to investigate the mechanism by which palmitate causes this dysfunctional differentiation, we next examined the insulin signalling pathway in differentiated and differentiating SH-SY5Y cells. Several animal and human studies have demonstrated an induction of brain insulin resistance in T2DM [39–41] and potentially LOAD, and previous studies have shown induction of insulin resistance in SH-SY5Y cells by high-level palmitate exposure [42]. Therefore, we tested the insulin signalling pathways using Western blotting for phospho-Akt and phospho-ERK. Firstly, total Akt levels in SH-SY5Y cells were reduced by chronic palmitic acid treatment, although this reduction was not significant (Fig. 3A, C). However, levels of phospho-Akt were significantly reduced at both D7 and D10 of differentiation, implying a reduction of tonic Akt activity induced by palmitic acid (Fig. 3B, C). In order to characterise insulin-responsive Akt phosphorylation (and therefore insulin signalling), Western blots of p-Akt were performed on cells with and without a 30-min 100 nM insulin stimulation (Fig. 3D). Our data indicates that, when differentiated in the presence of either 20 µM oleic or palmitic acid, SH-SY5Y cells display a complete loss of insulin-induced Akt phosphorylation, which we interpret as insulin resistance. As insulin signalling involves both the Akt and ERK pathways, we next investigated whether insulin-responsive ERK phosphorylation was similarly affected (Fig. 3E). Interestingly, and in contrast to some previous reports [42], we also noted a complete loss of insulin-responsive ERK phosphorylation in palmitate-exposed cells (Fig. 3E). Again, we also observed a similar reduction in insulin response in oleate-treated cells (although there was a non-significant trend to a higher level of ERK phosphorylation in oleate- vs. palmitate-exposed cells). Taken together, these data imply that chronic exposure of differentiating SH-SY5Y cells to clinically relevant levels of fatty acids induces profound dysfunction of insulin signalling via both the Akt and ERK pathways. Furthermore, this insulin resistance is induced by both saturated and unsaturated fatty acids, contrary to some previous observations, e.g. [42]. There are several possible explanations for this, but our contention is that the long-term, chronic exposure we have used during differentiation induces more physiologically relevant effects than the short-term, acute exposure employed by most studies. Given that the differentiation effects seen in Fig. 1 were mostly in response to palmitate, it is clear that chronic fatty acid exposure has multiple effects on differentiating neuronal cells, some of which may be mediated via insulin resistance.

**Fig. 4:**
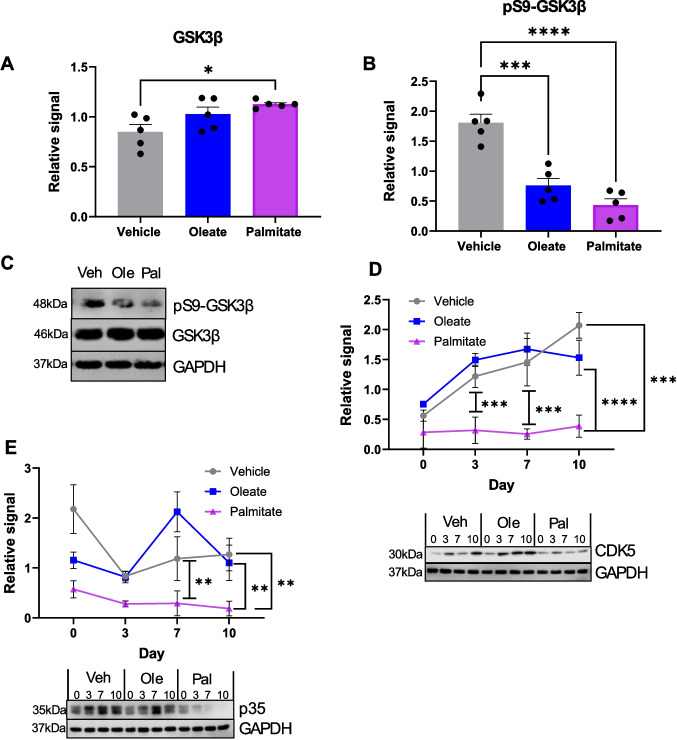
Chronic palmitate exposure during differentiation disrupts both GSK3β and CDK5 signalling in SH-SY5Y cells. **A**–**C** Western blotting quantification of GSK3β and GSK3β pS9 in D10 SH-SY5Y cells differentiated in the presence of 20 µM BSA-conjugated palmitic or oleic acid, or an equivalent volume of vehicle. All protein levels are normalised to a GAPDH loading control and normalised within each repeat. All values are the mean of four independent repeats, ± SEM. ***p < 0.001, ****p < 0.0001, 1-way ANOVA with Bonferroni’s post-hoc test. **A** Total GSK3β. **B** GSK3β phosphorylated at Ser-9 (pS9 GSK3β), normalised to GSK3β levels. **C** Representative Western blots. **D**, **E** Time course of CDK5 and p35 expressing during differentiation of SH-SY5Y cells under chronic oleate, palmitate or equivalent vehicle exposure. All protein levels are normalised to a GAPDH loading control apart from in **C** and normalised within each repeat. All values are the mean of four independent repeats, ± SEM. **D**, **E** Representative Western blot is shown below each graph. *p < 0.05, ***p < 0.001, 2-way ANOVA with Bonferroni’s post-hoc test

In order to investigate which other pathways could potentially be involved in our identified effects of free fatty acids on differentiation, we examined glycogen synthase kinase beta (GSK3β) and cyclin-dependent kinase 5 (CDK5). Both of these kinases have critical functions in neurogenesis [43–45] and, importantly, are implicated in the pathology of AD [5, 46–48]. Firstly, we examined GSK3β levels and phosphorylation at D10 of differentiation. We observed a small but significant increase in total GSK3β expression on palmitate exposure (Fig. 4A, C); however, the level of phosphorylation was dramatically and significantly decreased by both oleate and palmitate (Fig. 4B, C). As GSK3β is a well-described Akt substrate, this result is consistent with our previous data. Unlike Akt, GSK3β is inactivated by phosphorylation, suggesting that FFA exposure greatly increases GSK3β activity in this stage of differentiation. GSK3β has been reported to have different roles in different stages of neuronal differentiation, including synaptogenesis [44, 49, 50], and therefore, this change in GSK3β activity may be partly responsible for the differentiation defects we observe. As mentioned previously, GSK3β is known to be at least partially responsible for the hyperphosphorylation of tau in AD, suggesting that FFA exposure during differentiation may be an early event in tau pathology. Conversely, expression levels of both CDK5 and its activating subunit, p35, were significantly decreased in palmitate, but not oleate-exposed cells at every timepoint assayed during differentiation (Fig. 4D, E), demonstrating that palmitate exposure specifically reduces CDK5 expression and activity. Given the well-described role of CDK5 in neuronal function, differentiation and neurite outgrowth [45, 51], it is possible that a reduction in CDK5 activity, combined with the dysregulation of GSK3β, could potentially explain many of the developmental effects noted in Fig. 1. However, this warrants further investigation to fully clarify these mechanisms.

**Fig. 5:**
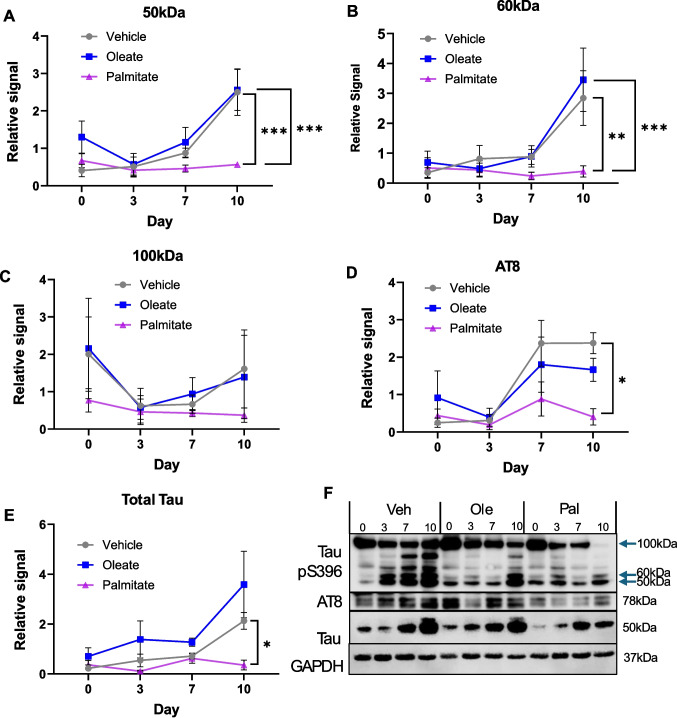
Chronic palmitate exposure decreases tau phosphorylation and expression. **A**–**C** Western blotting quantification of different molecular weight bands detected by tau pS396 antibody (**A** 50 kDa, **B** 60 kDa, **C** 100 kDa), at D0, D3, D7 and D10 SH-SY5Y cells differentiated in the presence of 20 µM BSA-conjugated palmitic or oleic acid, or an equivalent volume of vehicle. **D** Similar time course profile for AT8 phospho-epitope (pS202, pT205). **E** Total tau. **F** Representative Western blots. All protein levels are normalised to a GAPDH loading control and normalised within each repeat. All values are the mean of four independent repeats, ± SEM. *p < 0.05, **p < 0.01 ***p < 0.001,, 2-way ANOVA with Bonferroni’s post-hoc test

Given these changes in kinases associated with tau pathology, we next examined how FFA exposure affected phosphorylation of tau at 2 disease-associated epitopes: S396 and the AT8 site (S202 + T205). Interestingly, despite the increase we observed in active (unphosphorylated) GSK3β, chronic palmitate exposure reduced phosphorylation of tau at both the S396 and AT8 epitopes (Fig. 5A–D). For the pS396 blot, multiple bands were detected. The major bands were 50 kDa (which we assume to be single phosphorylation monomeric tau), 60 kDa (potentially multiply phosphorylated monomeric tau) and a band above 100 kDa (assumed to be dimeric/multimeric tau). All these bands decreased on palmitate exposure, although the decrease in the 100 kDa band lacked significance, likely due to high variability. Whilst both vehicle and oleate-exposed cells showed a time-dependent increase in phospho-tau during differentiation, this was completely absent from palmitate-exposed cells. On further analysis, this trend was likely due to the same effect on total tau levels (Fig. 5E)—whilst there was a robust increase in tau expression throughout differentiation in cells exposed to vehicle or oleate, this was absent from palmitate-exposed cells. Thus, palmitate exposure during differentiation inhibits tau expression, but the remaining tau does not appear to be phosphorylated at a higher level than control cells. Furthermore, as these effects are absent in oleate-treated cells, we assume that this reduction is not due to insulin resistance, which was induced by both oleate and palmitate (Fig. 3), but potentially by the dysregulation of other pathways identified including CDK5 and GSK3β (Fig. 4). This therefore provides further evidence that the effects of fatty acids on differentiation are complex and multifaceted.

From these data, therefore, it seems unlikely that FFA exposure during differentiation would lead directly to tau pathology. However, the lack of tau expression could explain some of the effect on differentiation in palmitate-exposed cells, e.g. the reduced expression of synaptic markers. Interestingly, we did not observe a significant change in phospho-tau (either AT8 or pS396) in hiPSC-derived cortical neurones exposed to 20 µM palmitate (Fig. S5B,C); however, there was a significant reduction in total tau expression, similar to that seen in SH-SY5Y cells (Fig. S5A). This implies an increase in the proportion of tau that is phosphorylated. Similar to Fig. 1, this demonstrates that 20 µM palmitate induces differentiation defects in both hiPSC-derived forebrain neurones and SH-SY5Y cells, although there are differences in their response. There are several potential reasons for this. Firstly, it is possible that the point at which we expose the hiPSC-derived neurones to palmitate (at the neuronal precursor stage) represents a later differentiation timepoint than D0 in our SH-SY5Y model. Indeed we observe a distinctly different pattern on the pS396-tau Western blot in hiPSC-derived neurones and SH-SY5Y cells (compare Fig. S5 with Fig. 5F). Secondly, it is possible that differences in media composition, cell number or receptor expression affect the response to fatty acids.

**Fig. 6:**
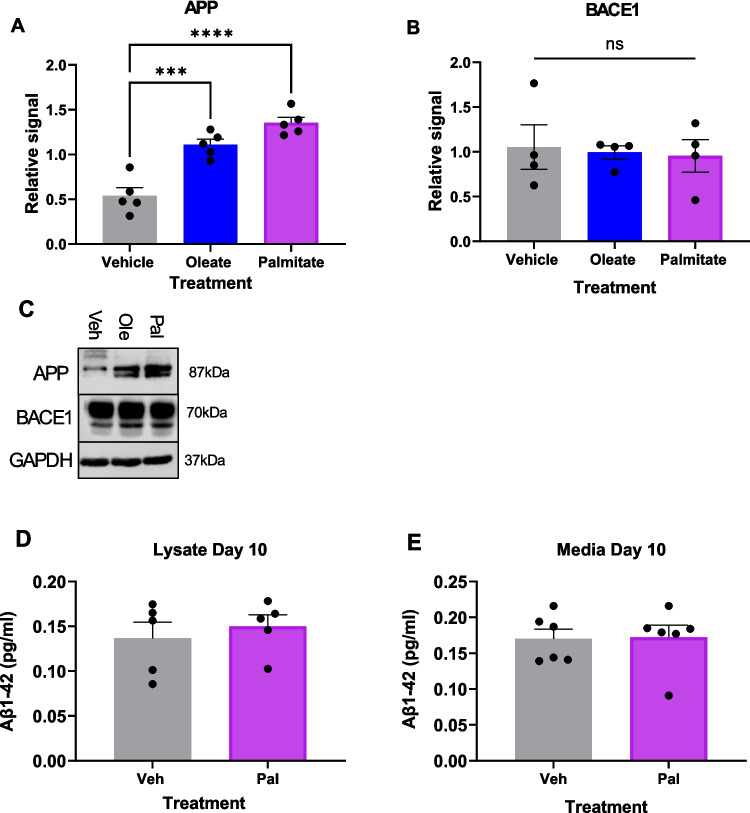
Chronic palmitate exposure increases APP expression but not Aβ production. **A**–**C** Western blotting quantification of APP (**A**) and BACE1 (**B**) in D10 SH-SY5Y cells differentiated in the presence of 20 µM BSA-conjugated palmitic or oleic acid, or an equivalent volume of vehicle, normalised to GAPDH levels and normalised within each repeat. ***p < 0.001 (1-way ANOVA). **C** Representative Western blots. **D**, **E** Amyloid beta ELISA performed on lysates (**D**) and media (**E**) from D10 SH-SY5Y cells differentiated in the presence of 20 µM palmitate or vehicle. ns, no significant difference, Student *t*-test

We next examined the effect of FFA exposure on beta-amyloid, the other major pathological hallmark of AD. We first examined expression levels of the amyloid precursor protein (APP), whose aberrant processing is thought to underlie Aβ deposition in the AD brain. SH-SY5Y cells differentiated in the presence of palmitate expressed significantly increased levels of APP (Fig. 6A, C), whereas oleate exposure led to a non-significant increase. However, this was not paralleled by the expression of the beta secretase, BACE1, an important enzyme in the amyloidogenic processing of APP [1] (Fig. 6B, C). Therefore, although palmitate exposure increased APP expression, this would not necessarily lead to a change in APP processing. In order to examine this, we used an ELISA assay for Aβ to examine secreted and intracellular Aβ levels. Surprisingly, given the large increase in APP levels observed on palmitate exposure, there was no increase in Aβ levels detected under any conditions, either in the cell lysates or the concentrated cell medium (Fig. 6D, E). We therefore conclude that dysfunctional neuronal differentiation triggered by chronic palmitate exposure does not lead directly to an increase in the two pathological hallmarks of AD. One potential explanation is that palmitate-induced neurogenesis defects may represent an early stage in the pathology of AD, in line with previous studies [22]. However, an alternative explanation is that SH-SY5Y cells produce only small levels of Aβ which is below the sensitivity range of the ELISA assay employed. Future studies will test this hypothesis using hIPSC-derived forebrain neurones. However, it should be noted that dysfunctional neurogenesis has been suggested to precede both amyloid and tau pathology [52]. Therefore it is possible that the defects in kinase signalling pathways we have identified could be a useful early indicator of LOAD.

We also performed Western blotting on hIPSC-derived forebrain neurones exposed to 20 µM palmitate throughout differentiation and noted no increase in APP expression (Fig. S6A); however, there was a small but significant increase in BACE1 expression (Fig. S6B). Combined with data from Fig. S5, this implies that exposure of differentiating hIPSC-derived neurones to 20 µM palmitate may induce changes consistent with AD pathology.

**Fig. 7:**
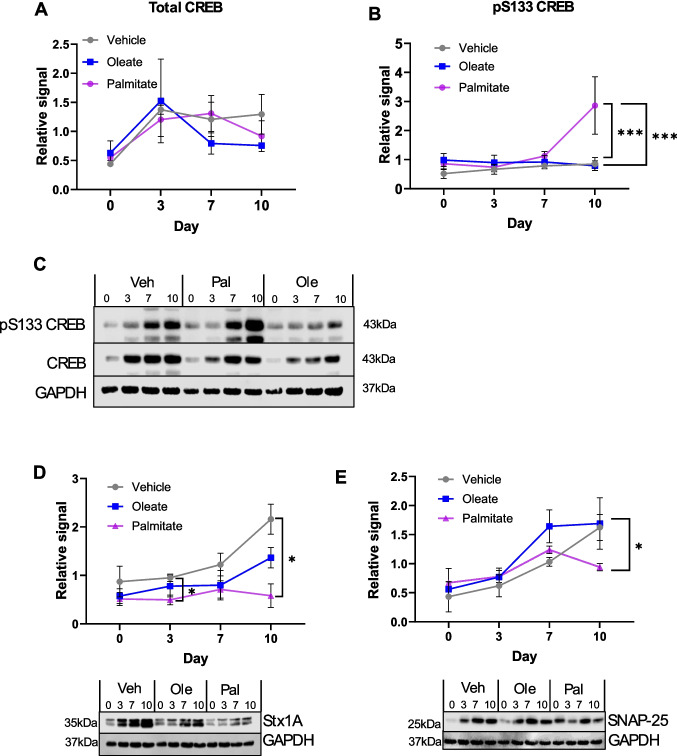
Chronic palmitate exposure during differentiation dysregulates phosphorylation of CREB and inhibits expression of key presynaptic proteins. **A**–**C** Western blotting quantification of CREB expression and phosphorylation in SH-SY5Y cells differentiated in the presence of 20 µM BSA-conjugated palmitic or oleic acid, or an equivalent volume of vehicle. **A** Total CREB1. **B** pS133 CREB1. **C** Representative Western blots. **D**, **E** Quantification of syntaxin1A (**D**) and SNAP-25 (**E**) expression in treated cells at D10, with representative Western blots shown below. All signals are normalised to a GAPDH loading control and normalised within each repeat. All values are the mean of four independent repeats, ± SEM. *p < 0.05, ***p < 0.001, 2-way ANOVA with Bonferroni’s post-hoc test

**Fig. 8:**
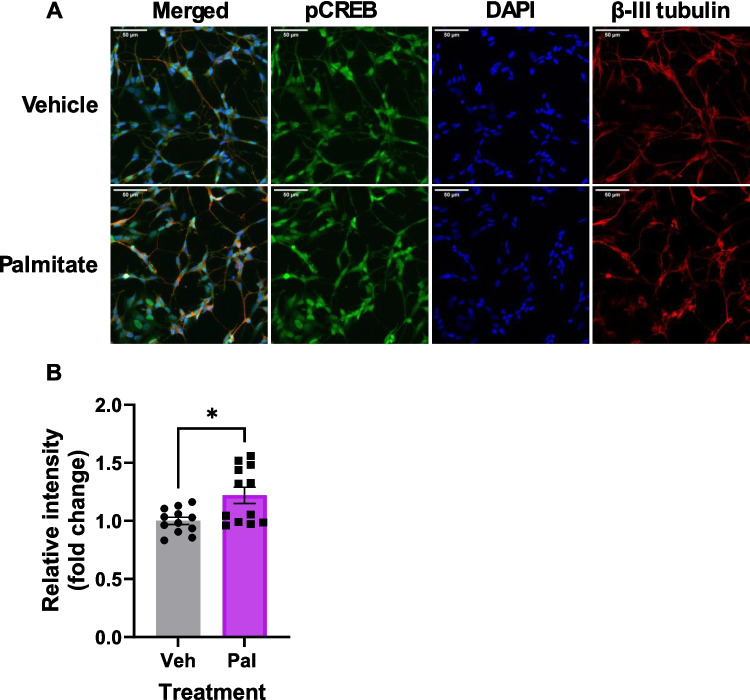
Chronic palmitate exposure during differentiation increases phospho-CREB nuclear localisation. **A** Representative immunocytochemistry images of phospho-CREB (green), β-III tubulin (red) and DAPI (blue) staining in D10 cells following chronic FFA treatment. Scale bar = 50 µM. **B** Quantification of nuclear phospho-CREB (DAPI-localised) signal (*n* = 12). *p < 0.05, Student *t*-test

In order to further investigate the mechanisms by which fatty acid exposure induces dysfunctional neuronal differentiation, we examined the phosphorylation of the key transcription factor, cAMP-response element binding protein (CREB). Once activated by PKA-mediated phosphorylation, CREB induces the transcription of several key genes involved in synaptogenesis and synaptic plasticity (for detailed review, see [53]). To our surprise, we observed a significant increase in CREB phosphorylation specifically in palmitate-treated SH-SY5Y cells (Fig. 7A–C). Given the defects in differentiation we observed on palmitate exposure, we would expect phospho-CREB to be reduced; however, given the important role for CREB phosphorylation in neuronal differentiation [54], it is entirely possible that inappropriate, excessive CREB phosphorylation could lead to dysfunctional differentiation. Indeed, aberrant over-phosphorylation of CREB has been observed in AD models [55]. For this reason, we examined the expression of key SNARE proteins known to be phospho-CREB targets, syntaxin 1A and SNAP-25. In line with dysfunctional synaptogenesis, we observed a decrease in the expression of both of these markers at D10 in response to palmitate exposure (Fig. 7D, E), indicating that this increase in phospho-CREB does not induce overexpression of CREB targets in our model system. The reasons for this are unclear, but we hypothesised that palmitate exposure might induce a mis-localisation of phospho-CREB away from the nucleus. We therefore tested this using immunocytochemistry, examining the relative intensities of nuclear (DAPI-localised) phospho-CREB in differentiated SH-SY5Y cells exposed to palmitate (Fig. 8A, B). However, this analysis revealed a significant increase in nuclear phospho-CREB in palmitate-exposed cells, consistent with our Western blotting data (Fig. 7). Interestingly, pathological overactivation of CREB has been noted in other neurodegenerative diseases, e.g. amyotrophic lateral sclerosis (ALS), and this was associated with reductions in synaptic proteins [56]. This therefore may represent a more general feature of neurodegeneration. We therefore conclude that aberrant, hyperphosphorylation of CREB may have a role in FFA-induced dysfunctions in neuronal differentiation; however, this merits further investigation.

**Fig. 9:**
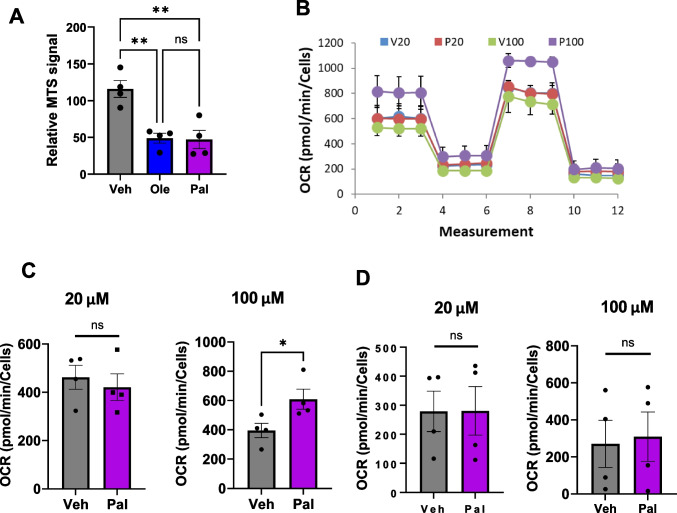
Chronic palmitate exposure during differentiation causes a dysregulation of metabolism in SH-SY5Y cells. **A** MTS assay performed on D10 differentiated SH-SY5Y cells, differentiated in the presence of 20 µM oleate, palmitate or vehicle control. Data are normalised to vehicle-treated cells, and presented as mean ± SEM. Three independent experiments each consisting of three technical repeats. **p < 0.01, 1-way ANOVA with Bonferroni’s post-hoc test. **B**–**D** Mitochondrial stress test using the Seahorse XFe24 assay of SH-SY5Y cells differentiated in the presence of 20 µM or 100 µM palmitic acid (*n* = 3). **B** Assay oxygen consummation rate (OCR) trace. **C** Basal metabolic rate. **D** Spare respiratory capacity, calculated as the difference between basal and maximal OCR. *p < 0.05, 1-way ANOVA with Bonferroni post-hoc test

Finally, given the effect we have observed on multiple signalling pathways, and the critical role of fatty acids in metabolism, we investigated the effect of differentiation in the presence of palmitate on mitochondrial metabolism in SH-SY5Y cells. Initial results showed a significantly decreased signal from an MTS assay in SH-SY5Y cells differentiated in the presence of palmitate (Fig. 9A). Whilst the MTS assay is frequently used as a measure of cell viability, the colorimetric signal generated results from the action of NADPH-dependent dehydrogenases (mainly mitochondrial succinate dehydrogenase) and, in the absence of decreased cell number in palmitate-treated cells (indicated by the consistent GAPDH signal in our loading control blots), may indicate a decrease in mitochondrial metabolic rate. We therefore investigated this using a Seahorse™ XFe24 Metabolic Analyser, using the mitochondrial stress test protocol. The results of this assay (Fig. 9B–D) indicate that none of the metabolic parameters was significantly impacted by 20 µM palmitate exposure. For clarity, we have only shown basal metabolic rate and spare metabolic capacity in this figure; however, the assay also measures proton leak and coupling efficiency, none of which was affected. In contrast, exposure to 100 µM palmitate during differentiation resulted in a significant increase in basal metabolic rate, but no other parameters. It should be noted that 100 µM palmitate resulted in a 40–60% decrease in cell number (as measured by crystal violet post-normalisation of the Seahorse assay). These results indicate that the levels of palmitate we have observed to disrupt SH-SY5Y cell differentiation have no overt effect on mitochondrial metabolism, and therefore, we presume that the effects of palmitate on the cells are mediated via another means. Possibilities include the fatty acid receptors GPCR40/120 [57], the nuclear receptor PPARγ [58] or ceramide production [59]. This, therefore, merits further investigation. Why the MTS assay result was reduced without a reduction in either cell number or metabolic rate is uncertain. It is possible that some cellular dehydrogenases are inhibited by palmitate; however, this is compensated for by other cellular enzymes.

## Conclusion

In this study, we have demonstrated a unique vulnerability of differentiating SH-SY5Y cells and neurones to chronic exposure to physiologically relevant levels of FFAs. Such exposure induces a wide-ranging dysfunction in differentiation and multiple critical signalling pathways. These include insulin signalling, GSK3β, CDK5 and CREB, although the significance of each of these is currently unclear and warrants further investigation. We therefore hypothesise that the chronic exposure of developing neurones in the brain to FFAs in T2DM explains at least part of the link between T2DM and late-onset AD and is consistent with decreased AHN in both AD and T2DM models. We do fully acknowledge that the SH-SY5Y model may not faithfully represent responses of neurones in vivo. In our SH-SY5Y cells, we do not see any increase in Aβ or p-Tau; however, we do not rule out the possibility that this is due to low Aβ processing in these cells. It is also clear that there are differences in the responses of SH-SY5Y cells and hIPSC-derived forebrain neurones (which potentially do show increases in phospho-tau and also enhanced BACE1 expression), although the principle of dysfunctional differentiation induced by chronic palmitate exposure still holds. Therefore, this warrants further investigation in a more representative cellular model, e.g. hIPSC-derived forebrain neurones, potentially with a comparison between different genotypes (e.g. ApoE4/4) or derived from healthy controls and AD patients.

The mechanisms behind this dysfunction are currently unclear and warrant further investigation; however, there are several potential signalling pathways which may be disrupted/activated by exposure to FFAs. As mentioned earlier, the fatty acid receptors GPCR40/120 have been shown to be activated by both saturated and unsaturated fatty acids [57] and have been associated with nervous system function and implicated in neurodegenerative diseases. Conceivably, overactivation of either of these GPCRs could account for the increase in p-CREB we observe due to their Gα_q_ linkage and would therefore increase intracellular Ca^2+^ once activated. Another potential pathway is the peroxisome proliferator-activated receptor (PPAR) family, particularly PPARγ. These fatty acid-binding nuclear receptors regulate the transcription of a variety of genes and have been implicated in neuronal differentiation and neurogenesis [58, 60]. Indeed, overactivation of PPARγ has been demonstrated to have deleterious effects on neural stem cells, consistent with our data. Additionally, ceramide production, for which palmitate availability is a rate-limiting step, is well established to adversely affect cellular signalling pathways, including that of insulin, via activation of protein phosphatase 2 A [61] and is additionally linked to AD pathology [59]. Therefore, it is possible that chronic palmitate exposure leads to ceramide accumulation, PP2A activation and disruption of multiple phospho-dependent signalling pathways which, in turn, leads to the disruption of differentiation we have observed.

Therefore, despite the unresolved questions, we believe that our data demonstrate a previously unexplored link between T2DM and AD which may have significance for the early pathology of this disease and offer potential therapeutic targets.

## Supplementary Information

Below is the link to the electronic supplementary material.

ESM 1 (DOCX 6.08 MB)

## Data Availability

Data is provided within the manuscript or supplementary information files.
