# Supplementary material for: Chronic Fatty Acid Exposure Disrupts SH-SY5Y and Neuronal Differentiation and Is a Potential Link Between Type-2 Diabetes and Alzheimer’s Disease
Source: Mol Neurobiol. 2025 Nov 27;63(1):183. doi: 10.1007/s12035-025-05367-6 (PMC12657545; doi:10.1007/s12035-025-05367-6)
Supplement: Supplementary file 1 — (DOCX 6.08 MB) [file 12035_2025_5367_MOESM1_ESM.docx]

# Chronic fatty acid exposure disrupts neuronal differentiation and is a potential link between Type-2 Diabetes and Alzheimer’s Disease

# **Supplemental Data**

## Authors:

Imogen L Targett^1^, Kate Pring^1^, Ana Martinez Valiente^1^, David Qualtrough^1^, Myra E Conway^2^ Lucy A Crompton^1^ and Tim J Craig^1^*

**Affiliations:**

^1^Centre for Research in Biosciences, School of Applied Sciences, University of the West of England, Bristol, BS16 1QY, UK

^2^University of Derby, Derby, DE22 1GB, UK

***Corresponding author:** Dr Tim J Craig, School of Applied Sciences, University of the West of England, Coldharbour Lane, Frenchay, Bristol, BS16 1QY, UK. [tim.craig@uwe.ac.uk](mailto:tim.craig@uwe.ac.uk)

**Submitted to *Molecular Neurobiology*, April 2025. Resubmitted July 2025.**

# Targett et al. - Figure S1

### Figure S1 - Chronic SFA exposure during differentiation does not change differentiation markers measured by qPCR

**A-C:** qPCR analysis of assay DCX (**A**), MALAT1 (**B**) and NeuN (**C**) mRNA expression on indicated days. * = p < 0.05, ** = p < 0.01, *** = p < 0.001, 2 way ANOVA with Bonferroni’s post-hoc test. Data shown at the mean +/- SEM (n=3).

# Targett et al. - Figure S2

###

### Figure S2 - Chronic FFA exposure during differentiation causes lipid droplet accumulation in SH-SY5Y cells

**A-C**: Representative ICC images showing DAPI (blue), β-III tubulin (green) and LipidSpot610 (red) staining of D10 differentiated SH-SY5Y cells. **A:** Vehicle. **B:** 20 µM oleate. **C:** 20 µM palmitate. **D:** Quantification of lipid droplet signal, normalised to average vehicle values (n=12). *** = p < 0.001, one-way ANOVA with Tukey post-hoc test.

# Targett et al. - Figure S3

###

### Figure S3 – Post-differentiation exposure to 20 µM FFAs has no effect on synaptic markers in SH-SY5Y cells

**A:** Western blot analysis of synaptophysin in D10 SH-SY5Y cells exposed for 72h to 20 µM palmitate (representative blot below). **B:** Western blot analysis of PSD-95 in D10 cells exposed for 72h to 20 µM palmitate (representative blot below).

# Targett et al. - Figure S4

### Figure S4 –8uM FFAs have no effect on differentiation of SH-SY5Y cells

**A,B:** Western blotting quantification of key differentiation markers at 4 timepoints (D0, D3, D7 and D10) during SH-SY5Y cell differentiation, treated throughout the protocol with either 8 µM BSA-conjugated palmitic or oleic acid, or an equivalent volume of vehicle. All protein levels are normalised to a GAPDH loading control and normalised within each repeat. All values are mean of 4 independent repeats, +/- SEM. A representative Western blot is shown below each graph. * = p < 0.05, ** = p < 0.01 (comparing palmitate treatment to control), 2-way ANOVA with Bonferroni’s post-hoc test. **A:** β-III tubulin. **B:** Synaptophysin. Note both proteins were visualised on the same blot therefore GAPDH loading control is the same for both.

# Targett et al. - Figure S5

### Figure S5 – Palmitate treatment of differentiating hiPSC-derived forebrain neurones decreases tau expression but not phosphorylation

**A-C:** Western blot analysis of total tau (**A**), pS396 tau (**B**) and AT8 (**C**) levels in D10 differentiated hIPSC-derived forebrain neurones exposed to 20 µM palmitate or equivalent vehicle control. Protein levels were normalised to GAPDH loading control and normalised within each repeat. Representative blots shown below graphs. Data are the mean of 3 repeats. * = p < 0.05, on Mann-Whitney unpaired t-Test.

# Targett et al. - Figure S6

###

### Figure S6 – Palmitate treatment of differentiating hiPSC-derived forebrain neurones increases BACE1 expression but not APP

**A,B:** Western blot analysis of APP (**A**) and BACE1 (**B**) levels in D10 differentiated hIPSC-derived forebrain neurones exposed to 20 µM palmitate or equivalent vehicle control. Protein levels were normalised to GAPDH loading control and normalised within each repeat. Representative blots shown below graphs. Data are the mean of 4 repeats. * = p < 0.05, on Mann-Whitney unpaired t-Test.
